# Unusual location of fibrous plaque in Indonesian child with tuberosclerosis complex

**DOI:** 10.11604/pamj.2017.28.92.12652

**Published:** 2017-09-29

**Authors:** Prastiya Indra Gunawan, Darto Saharso

**Affiliations:** 1 Department of Child Health, Airlangga University, Soetomo Hospital, Surabaya, Indonesia

**Keywords:** Tuberosclerosis, unusual location, fibrous plaque

## Image in medicine

Tuberosclerosis complex (TSC) is one of the most frequent genetic causes of epilepsy. A 3-year-old boy was reffered to Soetomo Hospital, Surabaya, Indonesia with chief complain of intractable seizures. The seizures were frequent and various in type. The skin present red dome shaped papules on face and smooth, firm, nodular or fleshy lesions referred to fibrous plaque on gum, right tarsal and right hallux region. Tarsal and hallux region were unusual skin collagenous fibroma area found in TSC. Five white macules with diameter 4-5mm were also found on the trunk. Electroencephalography resulted epileptiform discharges originating from left temporal posterior and right temporal. Head MRI showed multiple tubers at bilateral cortical-subortical fronto-temporo-parieto-occipital. The patient was diagnosed as tuberous sclerosis and received 3 types of anti epileptic seizure drugs (levetiracetam, carbamazepine and vigabatrin). The treatment resulted decrease of seizure frequency.

**Figure 1:**
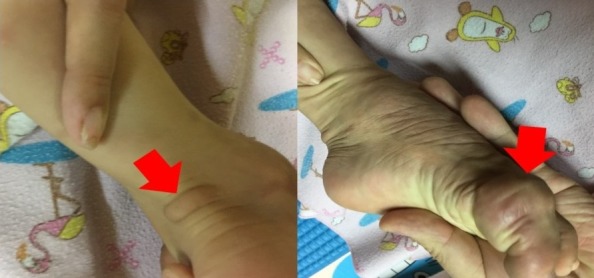
the unusual fibrous plaque in right tarsal and right hallux region

